# Numerical Study of Rice Grain Milling Uniformity in the Abrasive Milling Process

**DOI:** 10.3390/foods14040630

**Published:** 2025-02-13

**Authors:** Ze Sun, Anqi Li, Shouyu Ji, Hao Li, Zhuozhuang Li, Haonan Gao, Xinlei Wang, Xianle Li, Yanlong Han, Dan Zhao

**Affiliations:** 1 College of Engineering, Northeast Agricultural University, Harbin 150030, China; 13199519596@163.com (Z.S.); jiming@neau.edu.cn (A.L.); 15737200359@163.com (S.J.); 18686851312@163.com (H.L.); 18145122194@163.com (Z.L.); dbnydxghn@163.com (H.G.); wxl2801327323@163.com (X.W.); lxl000904@163.com (X.L.); 2 School of Water Conservancy & Civil Engineering, Northeast Agricultural University, Harbin 150030, China; dandanzhao_love@163.com

**Keywords:** rice milling quality, rice milling uniformity, abrasive milling process, parameter selection

## Abstract

The uniform removal of the bran layer significantly enhances the nutritional and economic benefits of rice. However, the influence of parameter conditions on the uniformity of milling in the abrasive milling process remains unclear. This is not conducive to improving the quality of rice milling. In this study, the effects of rotational speed and filling volume on milling uniformity in abrasive milling were investigated by combining experimental methods and simulation. The results showed that the higher the rotational speed, the more uniform the milling. The higher the filling volume, the more non-uniform the milling. The main reason for the variation in milling uniformity is the axial and radial position exchange of the rice particles in the milling chamber. The more frequent the exchange, the higher the milling uniformity. Subsequently, the frequency of position exchange was quantitatively characterised using axial and radial exchange rates, respectively. The rotational speed and filling volume change the position exchange frequency by affecting the rotational kinetic energy of the rice particles and the degree of dense rice population, respectively. These findings are useful in promoting rice loss reduction and nutritional balance.

## 1. Introduction

Over 50% of people worldwide eat rice as a staple food [1]. Research indicates that the bran layer serves as a significant source of dietary fibres, sterols, vitamins, and a range of antioxidants [2]. The nutrients found in rice bran are advantageous in reducing the risk of various illnesses, including cancer, hyperlipidaemia, and heart disease [3]. Therefore, people are gradually accepting moderately milled rice and even lightly milled rice [4]. The problem of precisely controlling the rice bran degree in the moderate or light milling process needs to be urgently solved. Since rice milling is a mass production procedure, milling uniformity becomes the key to precise retention of the bran. This is because improving milling uniformity can prevent a significant increase in breakage rates caused by over-milling and the excessive depletion of nutrients in the bran layer. This would greatly enhance the nutritional value and economic worth of the product. Abrasive milling is a rice milling process that offers a low broken rice rate and a more uniform removal of the bran layer. This characteristic makes the abrasive milling process suitable for the processing of suitable and lightly milled rice. Unfortunately, there is a lack of research specifically focused on the abrasive milling process. The underlying causes affecting the abrasive milling uniformity have not been clarified. This limits the further improvement of the abrasive milling uniformity. Therefore, this study aims to focus on comprehending the differences in uniformity during rice milling and enhancing it by optimising the relevant parameters.

Mohapatra investigated the effects of temperature rise and energy consumption on head rice yield in abrasive rice milling [5], as well as the application of the wear coefficient of rice grains to predict the degree of milling [6]. These studies demonstrate that researchers have developed a foundational understanding of the operational principles and methodologies of abrasive rice milling. However, research on milling quality remains relatively limited. As one of the critical parameters for evaluating the milling quality of rice grains, milling uniformity holds significant importance and warrants further investigation. Rice milling uniformity has been investigated in previous studies. Li et al. [7] proposed that the non-uniformity in friction milling stems from the existence of a blind zone in the milling process. They indicated that the uniformity of milling significantly depends on the capacity of rice to transition from the blind zones to the outer ring, quantifying this capacity through the alternating rate. An increased alternating rate correlates with improved milling uniformity. Fei et al. [8] identified the preferential orientation characteristics of rice grains as the cause of non-uniformity in friction milling. To summarise the above studies, it can be found that scholars have a more profound understanding of friction milling uniformity. However, there is a fundamental difference between abrasive and friction milling. Abrasive milling is predicated on the abrasive action of grits for bran layer removal, whereas friction milling depends on interactions among rice grains and between rice grains and the mill equipment under conditions of high pressure. Different working principles lead to different mechanisms of bran layer removal and different reasons for variations in milling uniformity. Therefore, investigating the abrasive milling uniformity is significant. Regardless of whether it is abrasive or friction milling, the uniformity of the milling process indicates how consistently bran is retained on the surfaces of all processed rice grains. Therefore, it is necessary to understand the rice bran layer removal process before exploring the milling uniformity of rice grains. Studies indicate that the removal of the bran layer throughout the milling process occurs in a systematic and staged manner. Ren et al. [9] proposed that the milling process can be categorised into four stages, namely, brown rice, seed coat crushing, grinding of the aleurone layer, and polishing of the endosperm. This fixed pattern of bran removal is related to the structure of the rice grain as well as the thickness and mechanical properties of the bran layer [10,11,12]. The variation in the milling uniformity of the rice grains is not associated with the bran layer removal mechanism; instead, it is most likely influenced by the parameter conditions of the milling equipment. Li et al. [13] examined how the filling level impacts milling uniformity, noting that as the filling level increased, the friction milling uniformity initially improved but then deteriorated. They proposed that the direct factor affecting milling uniformity was the rotational kinetic energy of the rice particles. Zhang et al. [14] indicated that the uniformity of milling is connected to the design of the rice mills. Specifically, in the left-hand or right-hand spiral configurations of the convex ribs, rice particles tend to accumulate at the inlet or outlet areas, respectively, resulting in non-uniform milling. Increasing the number of convex ribs can lead to better milling uniformity. In summary, previous scholars have suggested that milling uniformity is related to the structural and operational parameters of the rice mills. Therefore, it is necessary to explore the variation pattern of the abrasive milling uniformity under different parameter conditions. However, the specific parameter conditions affecting the abrasive milling uniformity are not yet clarified. Furthermore, a review of research indicates that modifications in the structural and operational parameters are generally associated with corresponding alterations in the flow patterns and kinetic properties of rice grains within the milling chamber. However, due to the sealed nature of the milling chamber, visual inspection of the rice grain flow is impractical. Additionally, the high rotational velocity of the milling rollers during the abrasive milling phase complicates the acquisition of kinematic and dynamic data on the rice grains through experimental means. These factors limit the in-depth exploration of abrasive milling uniformity.

Fortunately, benefiting from advancements in computer technology, numerical simulation techniques have become prevalent in agricultural engineering. Among these, the discrete element method (DEM) stands out as a favoured approach for addressing problems involving discontinuous media. Consequently, DEM has been extensively employed in particle flow modelling, demonstrating its effectiveness in capturing events on the particle bed and providing detailed flow insights [15,16]. Given its ability to extract granular data on particle velocity, force, and energy, DEM simulation is frequently utilised to study the motion characteristics of particles within diverse milling machinery, for example, the drum-type agitation mills [17], the ball mills [18], the vertical roller mills [19], and the vertical stirred mills [20], etc. Therefore, numerical analysis of the abrasive milling uniformity using DEM simulation is also considered in this study to obtain detailed microscopic information.

This study aims to investigate the variation in the abrasive milling uniformity under different parameter conditions. Consequently, our study focused on examining the correlation between milling uniformity and milling quality. The experimental data revealed the variation trends of milling uniformity across various rotational velocities and filling levels. Simulation techniques were utilised to dissect the flow patterns and kinetic properties of rice particles within the milling chamber under different parameter conditions. Ultimately, the study delved into the root causes behind the variation in milling uniformity. These findings are useful in improving the abrasive milling quality, thereby reducing losses and improving the nutritional value.

## 2. Materials and Methods

### 2.1. Experiment

#### 2.1.1. Experimental Equipment and Materials

**Figure 1:**
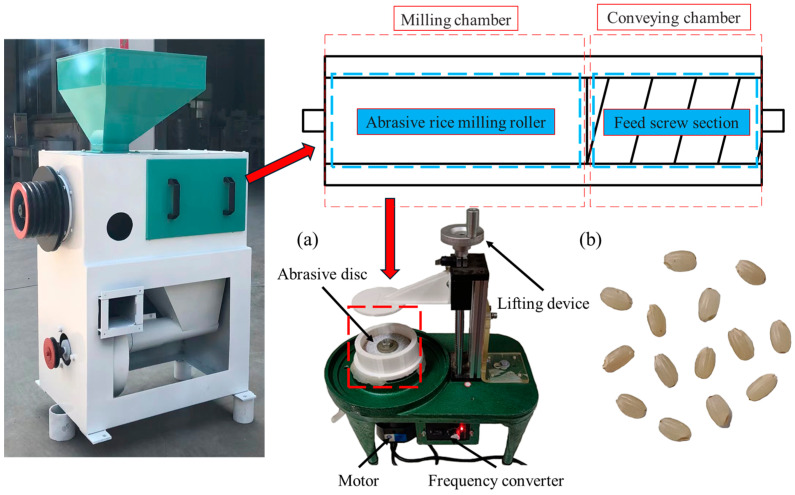
(**a**) Abrasive rice milling experimental platform, and (**b**) brown rice sample.

Traditional rice mills require a substantial amount of brown rice for processing. With flexibility and cost-effectiveness in mind for the experiment, a lab-scale milling setup was assembled, inspired by the operational principles and design of an abrasive rice mill. The configuration of the abrasive milling setup is depicted in Figure 1a. The setup comprises four primary elements, a 90 mm diameter, 60# sand disc which serves as the primary working component, a lifting device for sealing the machine casing, and a motor along with a frequency converter to regulate the rotation of the sand disc.

Japonica rice variety Suijing No. 18, sourced from Northeast Agricultural University in Harbin, China, was used for this study. The paddy with an initial moisture content of 11.37% (w.b.) was dehulled using a laboratory husker (model FC2K, Otake Company, Otake, Japan). The moisture content was determined using the standard oven-drying technique, with the oven maintained at a temperature of 105 °C for 3 h. After hulling, brown rice grains free from breakage or size irregularities were selected for further analysis, as illustrated in Figure 1b. To optimise the brown rice for milling, the samples underwent humidified and tempered processes to adjust their moisture content to the desired level (15.0 ± 0.5% (w.b.)). The prepared brown rice samples were then sealed and refrigerated at 4 °C for subsequent use.

#### 2.1.2. Rice Milling Experiment

Experiments on rice milling were undertaken to assess the uniformity of milled rice grains across various operational settings. Initially, the rice grains designated for milling are evenly distributed and smoothed out on the sand disc. Subsequently, the machine casing is sealed with the aid of the lifting device, and a frequency converter is utilised to modulate the rotational velocity of the sand disc. Upon powering the equipment and activating the switch, it commences operation. By manipulating the variables, a total of eleven distinct experimental setups can be configured, as detailed in Table 1. The milled rice samples are then preserved in airtight plastic containers and tagged for future experimental use.

#### 2.1.3. Sample Staining Experiment

**Figure 2:**
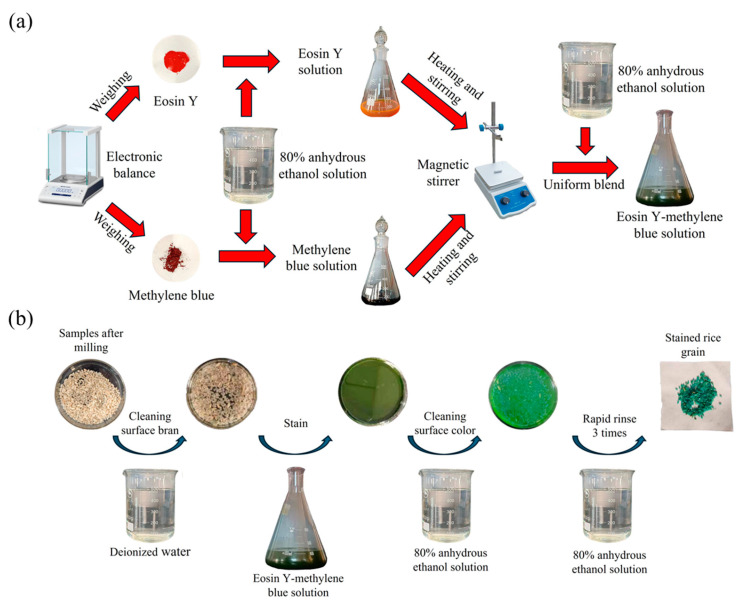
(**a**) The procedure for configuring eosin Y–methylene blue solution, and (**b**) the staining procedure.

The milled rice samples were treated with an eosin Y—methylene blue staining solution, which resulted in the endosperm taking on a purple-red hue and the bran layer appearing blue-green. This clear colour contrast between the endosperm and the bran layer aids in precisely determining the bran degree of the milled rice samples. The method for preparing the eosin Y—methylene blue solution and the staining process are depicted in Figure 2a and Figure 2b, respectively.

#### 2.1.4. Calculation of Uniformity

**Figure 3:**
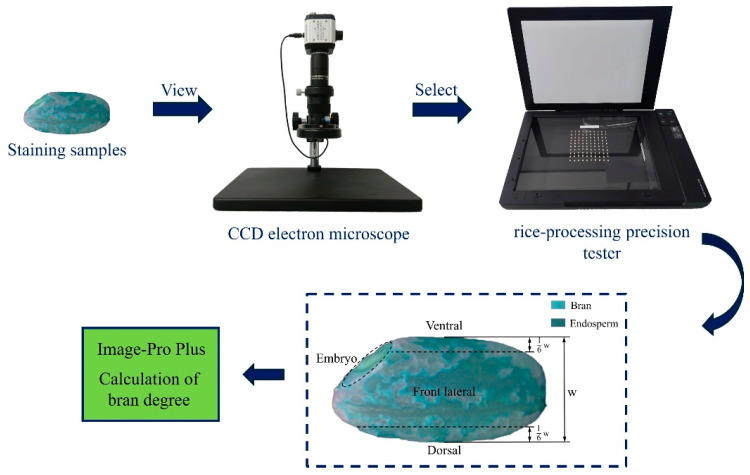
The method of calculating the bran degree of rice grains.

The milling uniformity (*UI*) is employed to assess the degree of uniformity in a batch of milled rice samples. It is calculated based on the variance of the bran degree, with a lower *UI* indicating a higher level of uniformity in the milling process. The formula for UI is as follows:

(1) $$UI=\frac{\sum_{i=1}^{N}{\left(Bd_{i}-\bar{Bd}\right)}^{2}}{N}$$

where *Bd* represents the bran degree, a measure of the extent to which the bran layer remains on the surface of the sample after milling; *Bd_i_* and $\bar{\mathrm{Bd}}$ represent the bran degree of sample *i* and the mean bran degree of *N* samples, respectively. The calculation of *Bd* is as follows:

(2) $$Bd=\frac{Sb}{S}\times 100\%$$

where *Sb* and *S* represent the area of the remaining bran layer and the scanning area of the milled rice, respectively. The milled samples underwent staining as described in Section 2.1.3. A random selection of fifty samples from the stained group was placed flat on the rice-processing precision tester (LBJD-A, Hangzhou Lvbo Instrument Co., Ltd., Hangzhou, China). The areas *Sb* and *S* were determined using Image-Pro Plus 7.1 software. The detailed procedure for this operation is depicted in Figure 3. To ensure the precision of the UI calculations, the process was repeated three times, and the mean UI value was computed. It should be noted that the rice grains were categorised into four distinct regions within the blue dashed square in Figure 3, with the back lateral region being the counterpart to the front lateral region, which is not visible in the figure.

#### 2.1.5. SEM Analysis

Surface microscopic details of the milled rice samples were captured utilising a scanning electron microscope (TM4000, Hitachi, Tokyo, Japan). The samples were prepared by adhering one surface to an aluminium plate using a conductive adhesive tape, while the opposite surface was coated with a layer of gold. The *SEM* was operated at an acceleration voltage of 15 kV to obtain the images.

#### 2.1.6. Acquiring the Motion Information of Rice

**Figure 4:**
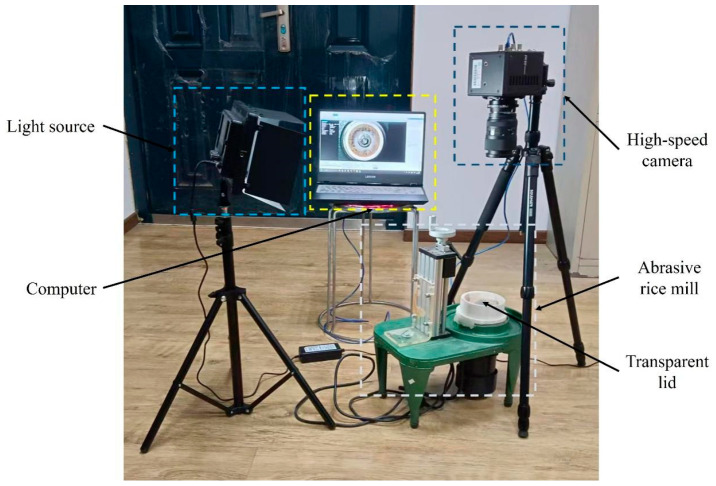
Image capture system for capturing the motion of rice grains.

The high rotational speed of the sand disc resulted in the high-speed motion of the rice grains. Acquiring information on the motion of rice grains is challenging. Therefore, an image acquiring system was constructed, which includes four parts, namely, an abrasive milling platform, a computer, a high-speed camera and a light source, as shown in Figure 4. It should be mentioned that the original cover of the milling chamber was substituted with a transparent acrylic one to allow for better visibility of the internal milling processes. Since the rice grains do not interact with the lid, their motion within the chamber remains uninfluenced.

### 2.2. Numerical Simulations

#### 2.2.1. Discrete Element Method

Because the milling chamber is completely closed, and the rice grains are in high-speed motion, it is impossible to obtain information on the kinematics and mechanics of the milling process by experimental methods. Fortunately, discrete element methods (*DEMs*) solve this problem. In this study, the commercial software EDEM™ (DEM Solution Ltd., Edinburgh, UK) was applied to simulate the milling process of rice grains in the abrasive milling platform. The software was installed on a computer equipped with an Intel Core 2 Duo processor, 8 GB of RAM, and a 64-bit Windows 10 Professional operating system. With this configuration, approximately 5.5 CPU hours were required to simulate 1 s of real-time particle movement.

This study employed a three-dimensional DEM approach based on the soft-sphere model. It is assumed that contact between rice grains does not involve cohesive forces and liquid bridges. Therefore, a non-sliding Hertz–Mindlin contact model was used to calculate the forces and torques between particles and geometry in the current study. The no-slip Hertz–Mindlin model, which combines Hertz’s theory in the normal direction and Mindlin’s no-slip model in the tangential direction, was employed in modelling each contact between particles or particle and geometry within the software EDEM. The trajectory of each particle is governed by Newton’s second law of motion, and the relevant equations that dictate their behaviour are as follows:

Translational motion:

(3) $$m_{i}\frac{dv_{i}}{dt}=m_{i}g+\sum_{j=1}^{n_{i}}F_{ij}$$

Rotational motion:

(4) $$I_{i}\frac{d\omega_{i}}{dt}=\sum_{j=1}^{n_{i}}\left(T_{t}+T_{r}\right)$$

where $m_{i}$ is the mass of particle *i*; $v_{i}$ is the translational velocity; $I_{i}$ is the moment of inertia; $\omega_{i}$ is the angular velocity; *g* is the acceleration of gravity; $F_{\mathrm{ij}}$ represents the cumulative contact force between a particle and its adjacent particles or the rice mill itself; $n_{i}$ denotes the count of particles that are in contact with particle *i*; $T_{r}$ is the rolling friction torque; and $T_{t}$ is the tangential force torque. The overall duration of the simulation is set at 30 s, and the data storage interval is 0.01 s.

#### 2.2.2. Simulation Model of Geometry and Particles

**Figure 5:**
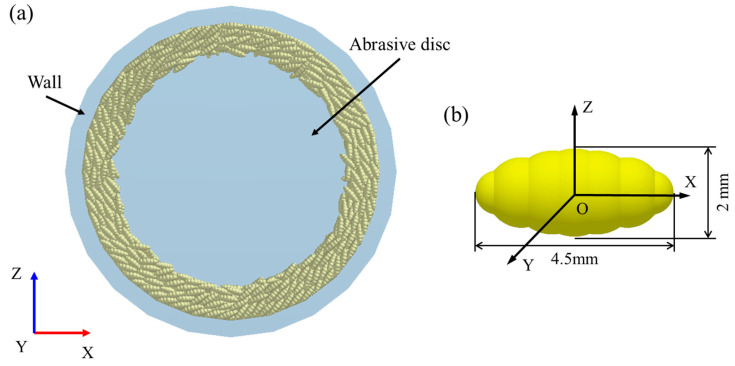
(**a**) Geometric model, and (**b**) particle model.

The geometric model was simplified to save simulation time. Only the critical components of the abrasive milling platform (including the sand disc, the machine body and the lid) were modelled, as shown in Figure 5a. The lid was hidden to demonstrate the motion of the rice grains. Rice particles can be modelled as axisymmetric ellipsoids. Non-spherical shapes like these can be represented using either the filling method or the multi-sphere approach in *DEM*. Although particles modelled using the filling approach will be closer to the real situation shape, the multi-sphere approach has a high contact efficiency. Therefore, the multi-sphere approach was selected for modelling rice particles in this study. It is worth noting that when using the multi-sphere approach, too few balls will result in a large difference from the real shape, and too many will result in a longer calculation time. Referring to previous studies, the rice particles were represented using the 7-sphere model as referenced in [7] and depicted in Figure 5b. The geometric parameters for the particle model were established by measuring the length, width, and height of a sample of 100 rice grains and then determining their mean values. Parameters encompassing material properties and contact characteristics for the simulation were sourced from experimental data and the existing literature, with a summary provided in Table 2 [7].

### 2.3. Model Validation

**Figure 6:**
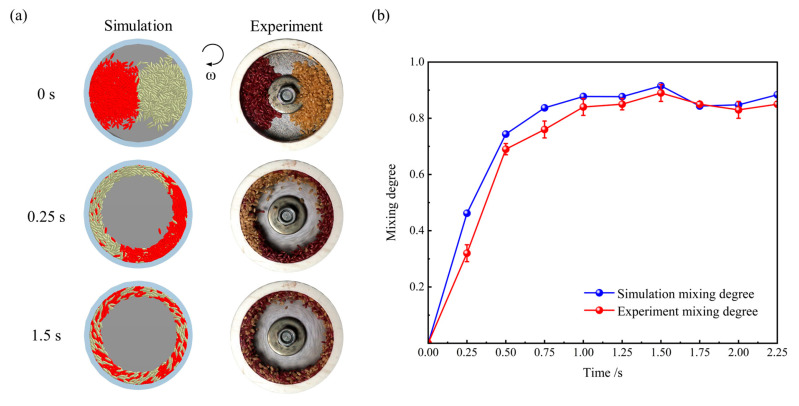
Comparison of simulation and experiment: (**a**) Snapshots of particles in motion, and (**b**) variation in MD with time.

Before analysing the simulation results, model validation experiments must be conducted to ensure the accuracy of the data. For mixing types of milling equipment, model validation is commonly performed by comparing the mixing degree (*MD*) in experiments and simulations [21]. Therefore, the effectiveness of the simulation model was verified by comparing the flow characteristics and mixing degree between the experiment and the simulation. *MD* serves as a measure of particle mixing uniformity, where values span from 0, signifying no mixing, to 1, representing perfect uniformity. The experimental data in this study can be acquired by the image acquiring system described in Section 2.1.6. Consider the parameter conditions of Case 1 as an illustrative example. Before the experiment started, 10 g of red-stained brown rice and 10 g of unstained brown rice were placed on a sand disc. The equipment was then activated to monitor the movement of the particles and track the temporal variation in *MD*. The formula for calculating *MD* is presented below:

(5) $$MD=1-\frac{\sigma }{\sigma_{0}}$$

(6) $$\sigma =\sqrt{\frac{1}{n}\sum_{k=1}^{n}{\left(r_{k}-r_{0}\right)}^{2}}$$

(7) $$r_{k}=\frac{N}{N_{T}}$$

where $\sigma_{0}$ is the initial value of σ; *n* is the number of the grid, the value of *n* is 16; $r_{k}$ denotes the proportion of red particles within grid *k*; $r_{0}$ signifies the proportion of red particles in a scenario of complete dispersion, which is 0.5; *N* and $N_{T}$ represent the count of red particles and the total particle count in grid *k*, respectively. The outcomes of the validation experiment are depicted in Figure 6. Figure 6a displays the snapshots of the simulation and the experiment at 0 s, 0.25 s and 1.5 s. Figure 6b shows the variation in MD with time. The results showed that the motion state of the particles and the trend of MD with time were similar in the simulation and experiment. This demonstrated that the simulation model was valid. However, the *MD* value of the experiment is smaller than the simulation in Figure 6b. This is attributed to a certain amount of time required for the motor to attain the predetermined rotational speed during the experiment. Moreover, although the rice grains were selected for minimal size variation in the experiment, they cannot be entirely identical, whereas the particles in the simulation were completely uniform in shape and size. Given that the simulation represents an idealised state, the difference is within the acceptable range.

## 3. Results

### 3.1. The Abrasive Debranning Process

**Figure 7:**
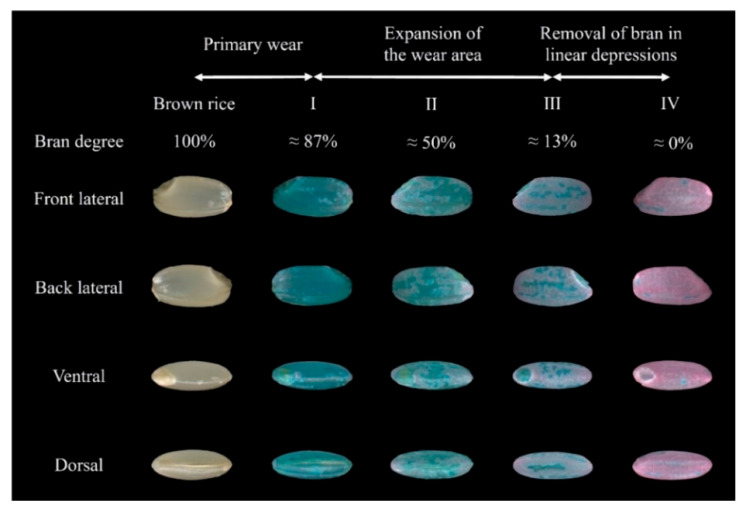
The process of rice bran removal in an abrasive rice mill.

To elucidate the impact of parameter settings on the milling uniformity in abrasive milling, it is essential to clarify the abrasive debranning process. Rice milling experiments were executed following the scheme detailed in Section 2.1.2. The samples were then stained after milling for observation and image scanning. Selecting typical surface features labelled as knots I, II, III, and IV, the images were sequenced according to milling time, illustrating the bran removal process in abrasive rice mills as depicted in Figure 7. Based on the different surface characteristics, the milling process can be summarised in three stages, namely, the primary wear stage (Brown rice—Knot I), the expansion of the wear area stage (Knot I–Knot III), and the removal of bran in the linear depressions stage (Knot III–Knot IV). During the initial phase, the rice grains experience surface damage, which is classified as the primary wear area. Progressing to the second phase, the bran layer of the rice is progressively stripped away as the wear areas enlarge. An approximate degree of damage to the bran layer on the lateral, dorsal, and ventral parts of the rice grains occurs during the milling process. In the final phase, the bran layer is incrementally eliminated from the linear grooves of the rice grains.

Upon examining the process of bran removal, it becomes evident that the development of wear areas plays a crucial role in the removal of the bran layer in abrasive rice mills. In other words, achieving a higher degree of milling uniformity entails that all rice grains undergo the same milling stage at the same time, thereby ensuring that the number and dimensions of the wear areas on each grain are nearly identical. However, this situation only exists in ideal conditions, as rice milling operates within the realm of mass production. Poor milling uniformity can lead to different degrees of bran removal from the same batch of rice grains at the same milling time. This issue can only be addressed through either repeated milling cycles or by prolonging the duration of the milling process. However, additional processing may lead to a substantial increase in energy usage and the likelihood of particle breakage. Therefore, enhancing the milling uniformity can be considered by adjusting the operational parameters of the equipment. The main parameters of the abrasive rice mill include the rotational speed, the grit size, and the rice grain fill level in the milling chamber. In actual production, the grit size can only be changed by replacing the sand discs or sand rollers. This approach is both labour-intensive and uneconomical. Therefore, only the effects of rotational speed and filling volume are considered in this study. We will explore the variation patterns of mill uniformity at different rotational speeds or fill volumes in the next section.

### 3.2. The Variation Pattern of Milling Uniformity

**Figure 8:**
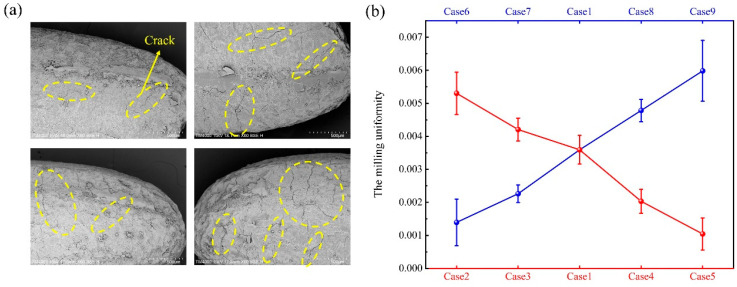
(**a**) SEM images of endosperm surface cracks in the removal of bran in linear depressions stage, and (**b**) the variation pattern of milling uniformity under different operating conditions.

The duration of the milling process is a critical factor in rice milling. Over-milling can lead to excessive removal of the bran layer, making it difficult to evaluate the uniformity of the milling. Conversely, under-milling might result in unstable operation of the equipment, which can compromise the accuracy of the experimental outcomes. Furthermore, even at the same milling duration, the degree of milling of rice grains can vary based on different parameter settings. To maintain the consistency of experimental variables, it is essential to establish the optimal milling time under various parameter settings before investigating the pattern of milling uniformity variations. The milling time for each set of samples can be ascertained by achieving the same bran degree. It should be observed that the sample surface after knot II exhibits a considerable amount of cracking attributable to fatigue wear, as illustrated in Figure 8a. Cracked rice grains are prone to break in the subsequent stages of processing. In summary, the milling uniformity of different experimental sets was compared at 50% bran degree in this study. The result of the comparison is shown in Figure 8b. The results show that the uniformity of the milling process tends to rise as the rotational speed increases and tends to fall as the filling volume increases. However, the cause behind this trend remains obscure. The discussion in Section 3.2 demonstrated that the mechanism of rice bran removal in abrasive mills remains consistent and is not influenced by variations in rotational speed or filling volume. Therefore, we hypothesise that alterations in the parameters might have resulted in modifications to the dynamic behaviour of the rice particles within the milling chamber, consequently affecting the milling uniformity. To prove this conjecture, the motion characteristics of the rice particles in the milling chamber are analysed in the next section.

### 3.3. Motion Characteristics of Rice Particles at Different Rotational Speeds and Filling Volumes

#### 3.3.1. Flow Structure of Rice Particles in the Milling Chamber

**Figure 9:**
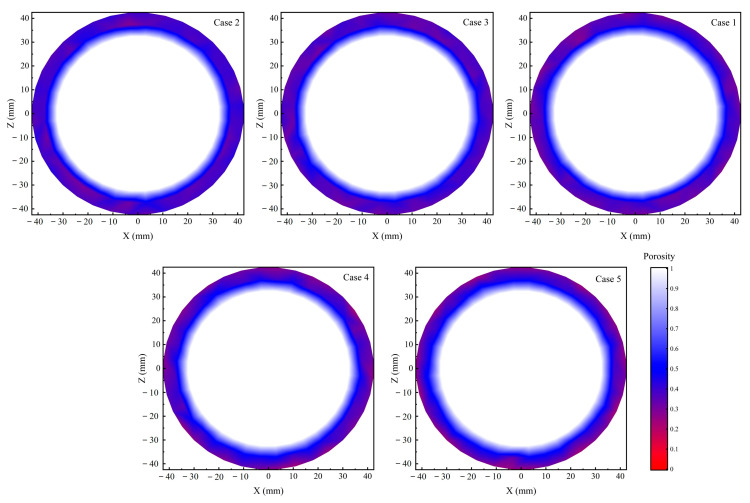
The spatial distribution of porosity in the XZ plane at different rotational speeds.

**Figure 10:**
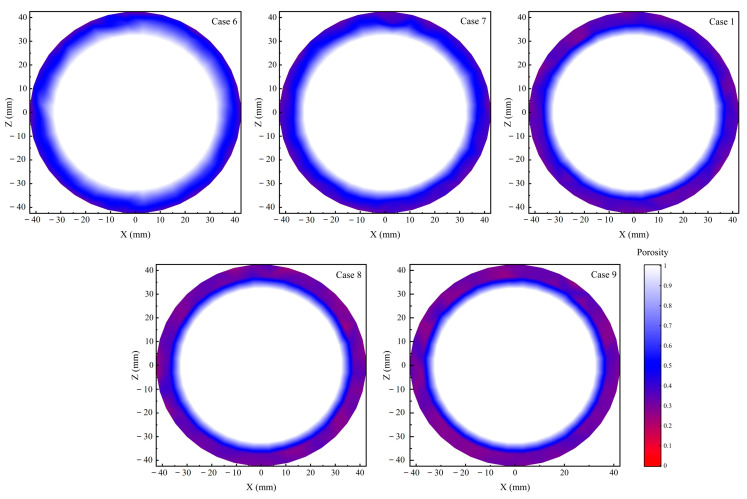
The spatial distribution of porosity in the XZ plane at different filling volumes.

Firstly, it is necessary to clarify whether the flow structure of rice grains is changed at different speeds and filling levels. To describe the particle flow structure, the spatial distribution of the porosity of rice particles in the XZ plane was calculated. Porosity (*P*) is commonly used to evaluate the concentration of particles at different positions. The smaller the value of porosity, the denser the particles at that position. The formula for calculating *P* is presented below:

(8) $$P=\frac{Vu-Vp}{Vu}$$

where *Vu* represents the volume of the cell; *Vp* represents the total volume of particles in the cell. In this study, the milling chamber was divided into 288 cells. The position of each cell is determined by the coordinates of its geometric centre. It is worth noting that particles can be distinguished by the centre of mass position when they are present in several cells simultaneously. Moreover, differences in the rotational speed of the sand disc across distinct experimental schemes can affect the accuracy of the calculated results. To eliminate errors, data were extracted for a period corresponding to four complete revolutions of the sand disc, starting at 5 s. Subsequently, the average was calculated. This approach makes the results more statistical. The same data extraction method is used in the later section and will not be repeated. Figure 9 and Figure 10 represent the spatial distribution of porosity in the XZ plane at different rotational speeds and filling volumes, respectively. Combined with the motion state of the particles in Figure 5a, it can be found that the rice particles undergo a circular motion in the milling chamber. Due to the high-speed rotation of the sand disc, the particles simultaneously form an annular and dense particle flow under the effect of centrifugal force close to the wall. Porosity values are smaller in positions close to the wall, and the particles are correspondingly denser. As the rotational speed increases, the spatial distribution of porosity changes little. As the filling volume increases, the particle porosity close to the wall increases significantly. In other words, variations in rotational speed have a minimal impact on the particle flow structure, whereas alterations in filling volume significantly influence the structure of the particle flow. The effect of filling volume on milling uniformity may be related to the variation in the flow structure. However, the specific reasons for the changes in milling uniformity are unclear. Further analyses are required to combine the working conditions of the abrasive rice mills and the motion characteristics of the rice particles.

#### 3.3.2. Motion Characteristics of Rice Particles in the Milling Chamber

**Figure 11:**
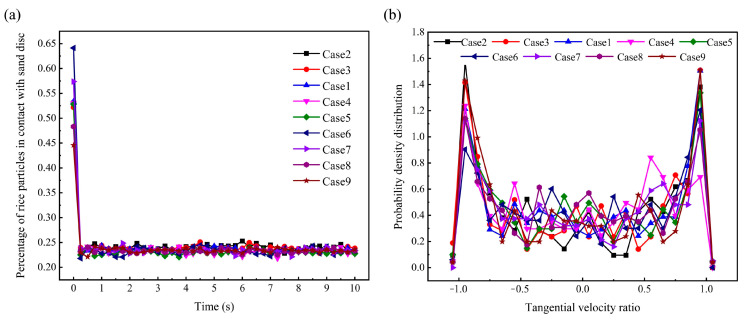
(**a**) The percentage of rice particles in contact with the sand disc, and (**b**) the probability density distribution of the TVR of the rice grains in contact with the sand disc under different parameter conditions.

Removing the rice bran layer is the wear process occurring on the surface of rice grains [6]. According to the Archard wear theory, the wear area is related to the relative motion between the two contacting objects and the material of the objects themselves. In this study, the objects in contact with the rice grains included the sand disc, other rice grains, and the machine body. This contact is negligible because the wall is sufficiently smooth. The sand disc is significantly harder than rice grains. Therefore, the contact between the rice grains and the sand disc is the main reason for the bran layer removal from the surface of the rice grains. Figure 11a shows the percentage of rice particles in contact with the sand disc for 0~10 s. It can be found that no matter how the equipment parameters are adjusted, the number of rice particles in contact with the sand disc under steady state is only 0.2 times the total number. This is caused by the annular flow structure of the rice grains in the milling chamber. In addition, the tangential velocities at different radius positions during the sand disc rotation are not completely the same. This phenomenon may result in a different trend of relative motion between the rice particles in contact with the sand disc and the sand disc, thereby resulting in a different wear degree of the rice bran layer. To analyse whether there is a difference in the relative motion between the rice particles and the sand disc, the concept of tangential velocity ratio (*TVR*) was proposed. The formula for calculating *TVR* is detailed below:

(9) $$v_{ta}=\omega \times r\times 0.1047$$

(10) $$TVR=\frac{v_{tr}}{v_{ta}}$$

where $v_{\mathrm{ta}}$ and $v_{\mathrm{tr}}$ represent the tangential velocity of the sand disc at any position and the tangential velocity of the rice particles at that position, respectively; ω is the angular velocity of the sand disc; *r* represents the distance from any position to the centre of the sand disc. When TVR is positive, the rice particles move in the same direction as the sand disc; when TVR is negative, the rice particles move in the opposite direction to the sand disc, and when TVR is 1, the rice particles are relatively stationary to the sand disc. Figure 11b shows the probability density distribution of the TVR of the rice grains in contact with the sand disc under different parameter conditions at simulation time 10 s. The results show that the trend of the probability density distribution of TVR under different parameter conditions is approximately the same, showing a double-peaked distribution between −1~1. It is not concentrated in a small range. Moreover, approximately half of the rice particles in contact with the sand disc move in the same direction as the sand disc, while the other half move in the opposite direction. This means that most of the rice particles at different positions have different trends of relative motion between the particles and the sand disc. Following the analysis, it is evident that significant issues exist within the process of bran layer removal in abrasive rice mills. On the one hand, only some of the rice particles can come into contact with the sand disc. On the other hand, the relative motion trends of the rice particles and the sand disc are different at different radial positions. Both result in different wear degrees on the same batch of rice grains, thereby failing to ensure the milling uniformity. Therefore, to ensure the milling uniformity, the rice particles in the milling chamber must break through the limitation of the position.

#### 3.3.3. Exchange of Axial and Radial Positions of Rice Particles in the Milling Chamber

**Figure 12:**
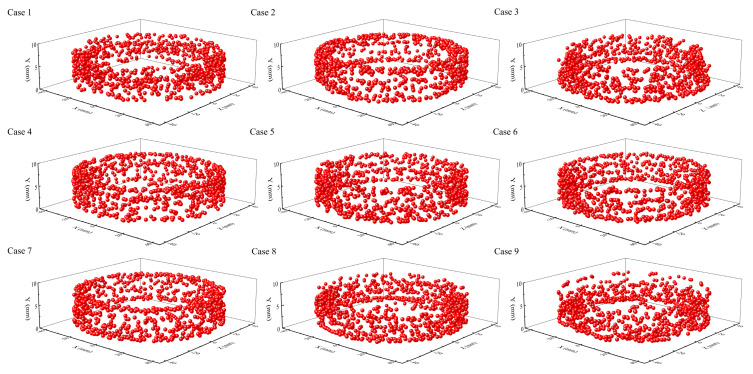
The trajectories of the tracer particles in the milling chamber from 0 to 10 s under different parameter conditions.

As mentioned in the previous section, to ensure uniform milling, the rice grains must change their spatial position. To prove this point, a tracer particle was randomly selected in the simulation with different parameter conditions, respectively. The trajectories of the tracer particles in the milling chamber from 0 to 10 s were subsequently analysed, as shown in Figure 12. It is worth noting that the time interval for extracting the position of the tracer particles was 0.01 s. In addition, to ensure clear observation of each instantaneous position, the trajectory of the tracer particles is expressed in the form of 3D points. It is readily observable that, irrespective of the parameter settings, the motion trajectories of the tracer particles are dispersed throughout the spatial domain of the milling chamber. Moreover, the 3D points approximately fill the interior of the annular band formed by the population of rice particles. This means that the spatial position of the rice particles is not constant during the milling procedure. Change in the position of each rice particle represents the motion of that particle from the initial position to another position in a certain time interval. This requires other rice particles to provide enough space for it. In other words, the spatial position of the other rice particles must change as well. If the process of motion is ignored, the position change can be understood as the rice particles exchanging position with other rice particles, including axial and radial position exchange. The exchange of radial positions avoids different wear degrees caused by different relative motion trends of the rice grains in contact with the sand disc. The exchange of axial positions ensures that every rice particle has the opportunity to come into contact with the sand disc.

**Figure 13:**
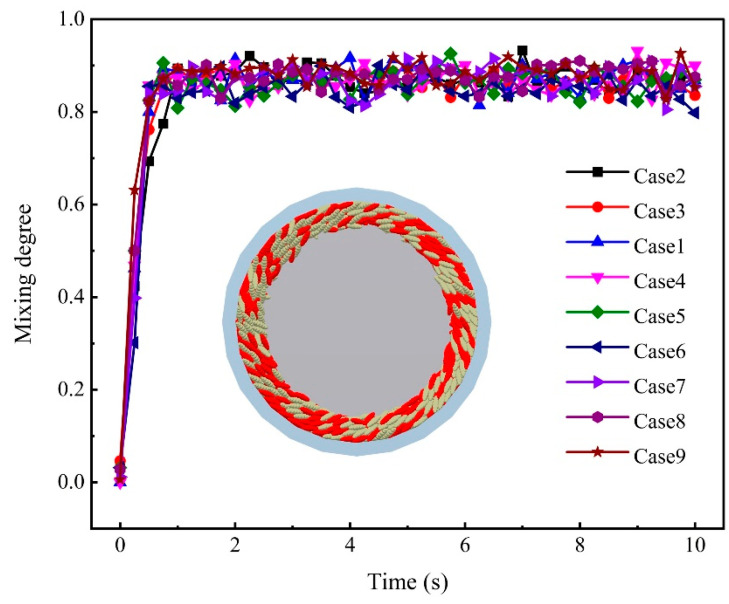
The variation in the mixing degree of rice particles with time under different parameter conditions.

To quantitatively verify the above conclusions, the *MD* of the rice particles under different parameter conditions was calculated according to the method in Section 3.1. The mixing of the rice particles actually refers to the fact that the particles undergo axial and radial position exchange. The higher the *MD*, the more intense the motion of position exchange between the rice particles. Therefore, we can consider applying the *MD* to predict the degree of position exchange of rice particles. Figure 13 shows the variation in the mixing degree of rice particles with time under different parameter conditions. The rice particles start to move steadily at about 0.5 s. The *MD* remains stable after 0.5 s. This phenomenon can be attributed to the period required for rice particles to transition from lying flat on the sand disc to forming an annular structure. Moreover, uniform particle mixing requires a certain period. The values of the *MD* for each case at steady state are greater than 0.8, which is close to complete mixing. This indicates that the particles undergo intense axial and radial position exchange in the milling chamber.

In summary, by interchanging the axial and radial positions of rice particles, the issues associated with the bran layer removal method are mitigated, ensuring a uniform level of wear across the rice grains. In addition, to improve the milling uniformity, the position exchange rate of rice particles must be increased. The more frequently the positions are exchanged, the more uniform the milling will be. From this, we speculate that the difference in milling uniformity under different parameter conditions is due to variations in rotational speed and filling volume affecting the position exchange rate of rice particles. To prove this conjecture, we will explore the effect of different parameter conditions on the position exchange rate of rice particles in the next section.

### 3.4. Effect of Rotational Speed and Filling Volume on the Position Exchange Rate

**Figure 14:**
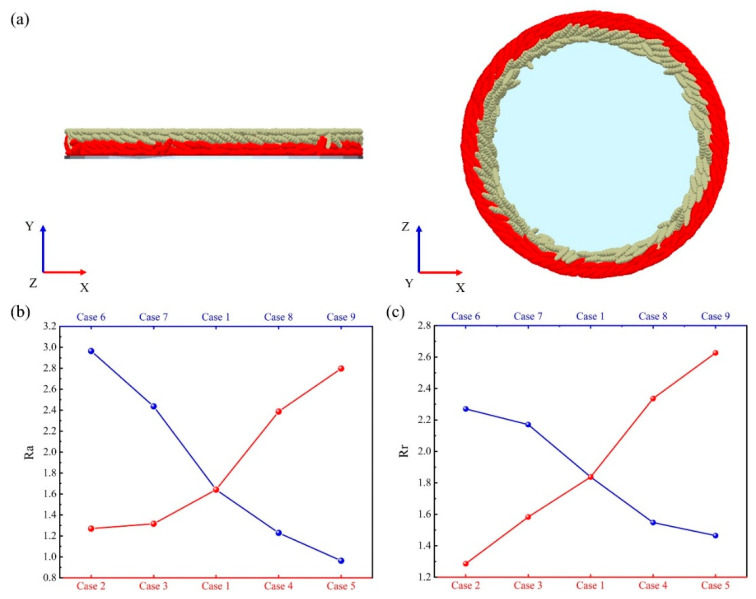
(**a**) Methods of marking bottom and outer circle particles, (**b**) axial exchange rates of particles at different rotation speeds and filling volumes, and (**c**) radial exchange rates of particles at different rotation speeds and filling volumes.

Firstly, the axial and radial position exchange rates of rice particles are required to be quantified, respectively. Referring to our previous research [13], the axial exchange rate *Ra* and radial exchange rate *Rr* are proposed. The specific calculations are as follows:

(11) $$Ra=\frac{Nb_{t_{0}}-Nb_{t_{0}+\Delta t}}{Nb_{t_{0}}\Delta t}$$

(12) $$Rr=\frac{No_{t_{0}}-No_{t_{0}+\Delta t}}{No_{t_{0}}\Delta t}$$

where $t_{0}$ represents the specified initial moment, and 5 s is specified as the initial moment in this study; ${\mathrm{Nb}}_{t_{0}}$ and ${\mathrm{No}}_{t_{0}}$ represent the number of particles marked in the bottom and outer circle at the initial moment, respectively; ${\mathrm{Nb}}_{t_{0}+∆t}$ and ${\mathrm{No}}_{t_{0}+∆t}$ represent the number of particles marked in the bottom and outer circle after the sand disc has rotated for ∆*t* s, respectively. It is worth noting that the variation in rotational speeds among various experimental schemes required employing different time intervals (∆*t*) for calculations. Therefore, ∆*t* in this study takes the time required for the sand disc to rotate 4 cycles in the different experimental schemes. The bottom and outer circle particles need to be marked before calculation. The particles in the range of 1 to 5 mm in *Y*-axis coordinates are the bottom particles, and particles in the range of 5 to 9 mm are the top particles. The particles in the radial position range of 35 to 40 mm are the inner circle particles and the particles in the range of 40 to 45 mm are the outer circle particles, as shown in Figure 14a. The results of the calculations are shown in Figure 14b,c. The results showed that as the rotational speed increased, the axial and radial exchange rate of rice particles rose, consequently enhancing the milling uniformity. The axial and radial exchange rates of rice particles diminished with an increase in filling volume, leading to a reduction in milling uniformity. This is consistent with the phenomenon observed in Figure 8b.

**Figure 15:**
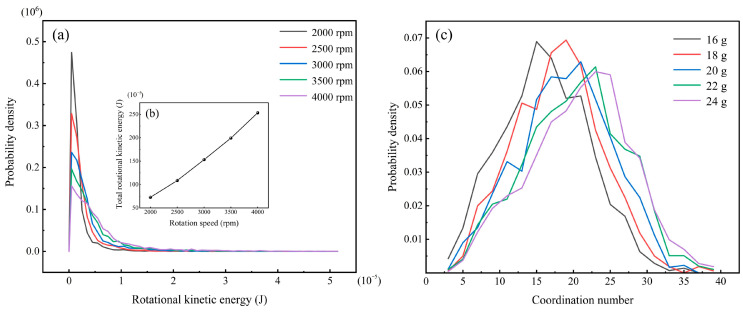
(**a**) Probability density distribution of particle rotational kinetic energy at different rotation speeds, (**b**) total rotational kinetic energy at different rotation speeds, and (**c**) probability density distribution of coordination number at different filling volumes.

Furthermore, it remains to be elucidated how variations in the rotational speed and the filling volume influence the axial and radial exchange rates of the rice particles. In a steady dense flow, the largest dimensions of non-spherical particles are aligned with a small inclination angle in the direction of the flow [22]. Therefore, the rice particles are approximately in the direction of the long axis and perform circular motion. If the rice particles always move in this preferential orientation, there is no possibility of axial and radial position exchange. The rotational kinetic energy of particles can be regarded as the kinetic energy that destroys the orderly motion of particles. In the actual milling process, the friction between the rice particles and the sand disc, the wall as well as between the rice particles is different. This results in relative motion between the grains of rice, which in turn leads to collisions. After the collision, the rice grains adjust their posture through rotation motion. Ultimately, the trend of orderly motion of the rice particle population is disrupted. When the rice grain rotates around the *Z*-axis, rice particles exhibit a propensity for radial position interchange. When the rice grain rotates around the *Y*-axis, the particles tend to axial position exchange. Figure 15a presents statistics on the probability density distribution of rotational kinetic energy of rice particles under different rotational speed conditions. It can be found that the peak of the distribution changed when the rotational speed was varied, which represents that the rotational kinetic energy of most of the particles would be affected by the external energy input. Moreover, with the increase in rotational speed, the distribution of rotational kinetic energy gradually becomes wider. In other words, as the rotational speed increases, the percentage of particles with high rotational kinetic energy increases. Meanwhile, the total rotational kinetic energy of the rice particles at different rotational speeds was counted as shown in Figure 15b. The results showed that with increasing rotational speed, the total rotational kinetic energy of the rice particles increased significantly. In other words, increasing the rotational speed of the sand disc enhances the rotational motion of the rice particles, thereby improving the capacity for axial and radial position exchange of the rice particles.

Summarising the above analysis, the intensification of the rotational motion of the rice particles only increases the trend of position exchange of the rice particles. Enough exchange space is still needed to complete the process. The dimension of the space available for position exchange is influenced by the filling volume. With the increasing filling volume of the rice particles, the space available for axial and radial position exchange of the rice particles gradually decreases. Figure 15c represents the probability density distribution of the coordination number of rice particles at different filling volumes, where the coordination number is defined as the number of contacts between each particle and the neighbouring particles, which varies to reflect the expansion and compaction of the particle bed. With increasing filling volume, the peak of the distribution gradually shifts to the right. In other words, as the filling volume increases, the denser the particle population becomes, the less space is available for the axial and radial position exchange of the rice particles.

## 4. Discussion

**Figure 16:**
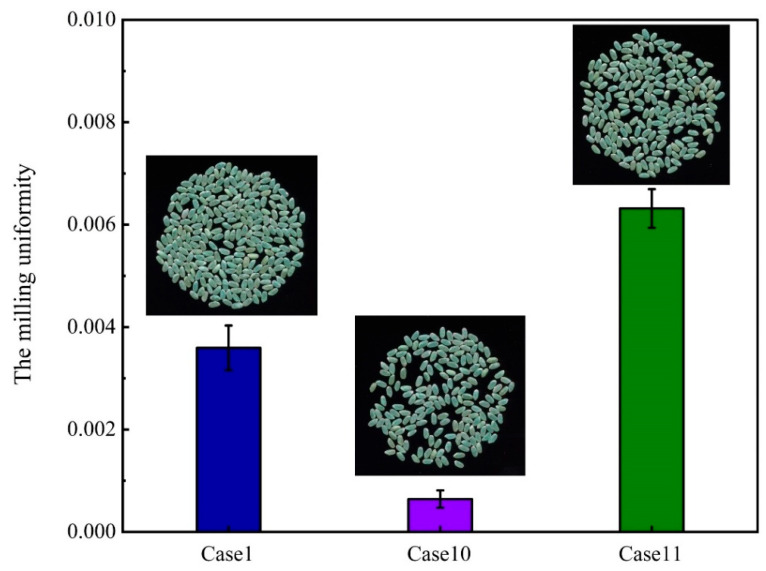
Comparison of milling uniformity in experimental scheme 2.

The above analysis shows that milling uniformity has a great influence on the milling quality of the abrasive rice mill. The higher the rotational speed, the more uniform the milling. The higher the filling amount, the more non-uniform the milling. To further verify this result, the milling uniformity of the rice grains was compared for case 1, case 10 and case 11 separately, and the results are shown in Figure 16. The milling uniformity of the rice grains was best in case 10, second in case 1 and worst in case 11. Therefore, focusing solely on milling uniformity, it is advisable to maximise the rotational speed and minimise the filling volume to the greatest extent feasible. Furthermore, simulation methods were applied to calculate the mixing degree or the position exchange rate to predict the milling uniformity under different parameter conditions. This method is simple, effective and economical. Contrasted with Li et al.’s [13] investigation into the milling uniformity of friction rice milling, this study has undertaken a comprehensive analysis of the pivotal factors influencing the milling uniformity of abrasive rice milling. The operational mechanisms of abrasive and friction rice milling are completely different. Therefore, this study is innovative and meaningful.

However, milling uniformity alone is insufficient as a criterion for assessing the overall quality of the milling process. Breakage rate, yield and energy consumption are equally important for the rice milling process. Excessive decrease in filling volume may result in lower yields. Excessively increasing the rotational speed not only increases energy consumption but also leads to higher breakage rates. However, this study only considers the milling uniformity of rice grains, and other indicators for evaluating milling quality are not taken into account. The benefits of improving milling uniformity by increasing the rotational speed and reducing the filling volume are likely to be offset. This is also a limitation of this study. Therefore, exploring the optimal parameter combinations for low energy consumption, low breakage rate, high yield and high milling uniformity is the next main goal for our research. Moreover, parboiled rice, renowned for its superior nutritional content, emphasises milling uniformity during processing to retain its nutrient profile and enhance overall quality. Consequently, future research could investigate the milling uniformity of parboiled rice using abrasive rice mills. Analysing the physical and structural properties of parboiled rice, along with optimising milling process parameters, could prove pivotal. This research direction has the potential to enhance the milling quality of parboiled rice and better preserve its nutritional components.

## 5. Conclusions

In this study, the removal process of the rice bran layer and the kinetic properties of the rice particles during the abrasive milling were examined by combining experimental methods and simulation. The effects of the rotational speed of the sand disc and the filling volume of the rice grains on the uniformity of abrasive milling were quantitatively and qualitatively analysed, and the underlying reasons for the effects were explained. The detailed findings are outlined below:

1. The uniformity of the milling process significantly impacts the quality of milling and is not contingent on the operational mechanics of the abrasive rice mills; instead, it is predominantly affected by the rotational velocity of the sand disc and the filling volume of the rice grains.
2. The axial and radial position exchange of rice particles is the main cause of variation in milling uniformity. The higher the exchange rate, the more uniform the milling. Mill uniformity can be predicted by the mixing degree or the position exchange rate.
3. The rotational speed of the sand disc increased, the rotational kinetic energy of the rice particles increased, the ability to disrupt the orderly motion of the rice particles increased, the axial and radial position exchange capacity of the rice particles increased, and the milling uniformity was improved.
4. As the filling volume of rice grains in the mill increases, their filling becomes more compact, consequently reducing the available space for positional interchange, the axial and radial position exchange capacity of rice grains is reduced, and the milling uniformity is decreased.
5. Focusing solely on milling uniformity, it is advisable to maximise the rotational speed and minimise the filling volume to the greatest extent feasible. However, a higher rotational speed will increase the energy consumption of rice milling and the broken rate of rice grains, and a lower filling volume will lead to a decrease in production. These factors may offset the benefits of improved milling uniformity.

## Figures and Tables

**Table 1:** Experimental scheme.

	Rotation Speed of Abrasive Disc (rpm)	Filling Mass of Rice Grains (g)
**Case 1**	3000	20
**Case 2**	2000	20
**Case 3**	2500	20
**Case 4**	3500	20
**Case 5**	4000	20
**Case 6**	3000	16
**Case 7**	3000	18
**Case 8**	3000	22
**Case 9**	3000	24
**Case 10**	4000	16
**Case 11**	2000	24

**Table 2:** Geometry parameters and physical parameters used in the simulation.

Type	Parameters	Value	Data Source
**Abrasive rice mills**	Radius × height (mm)	47.5 × 10	Own experiment
**Sand disc**	Radius (mm)	45	Own experiment
	Rotational speed (rpm)	2000~4000	
	Density (kg/m^3^)	2648	
	Poisson ratio	0.28	
	Shear modulus (Pa)	1.124 × 10^7^	
**Wall**	Density (kg/m^3^)	7800	Reference [7]
	Poisson ratio	0.3	
	Shear modulus (Pa)	7 × 10^8^	
**Rice particle**	Density (kg/m^3^)	1550	Reference [7]
	Poisson ratio	0.25	
	Shear modulus (Pa)	1 × 10^6^	
	Quality (g)	16~24	
**Particle–particle**	Restitution coefficient	0.68	Reference [7]
	Coefficient of static friction	0.15	
	Coefficient of rolling friction	0.01	
**Particle–wall**	Restitution coefficient	0.68	Reference [7]
	Coefficient of static friction	0.1	
	Coefficient of rolling friction	0.01	
**Particle–sand disc**	Restitution coefficient	0.45	Own experiment
	Coefficient of static friction	0.61	
	Coefficient of rolling friction	0.024	
**Simulation**	Time step(s)	1.5 × 10^−5^	

## Data Availability

The original contributions presented in the study are included in the article, further inquiries can be directed to the corresponding author.
